# Multi-Version Evaluation of Deep Learning Architectures for Tibial Plateau Fracture Detection and Deployment in a Web-Based Clinical Support System

**DOI:** 10.3390/diagnostics16193141

**Published:** 2026-09-27

**Authors:** Han-Ting Shih, Yuan-Hsin Sung, Endah Kristiani, Shun-Ping Wang, Chao-Tung Yang

**Affiliations:** 1 Department of Orthopaedics, Taichung Veterans General Hospital, Taichung City 407219, Taiwan; d13330003@thu.edu.tw; 2 Department of Industrial Engineering and Enterprise Information, Tunghai University, Taichung City 407224, Taiwan; 3 Department of Computer Science, Tunghai University, Taichung City 407224, Taiwan; g12350004@go.thu.edu.tw (Y.-H.S.); endahkristi@thu.edu.tw (E.K.); 4 Department of Informatics, Krida Wacana Christian University, Jakarta 11470, Indonesia; 5 Department of Post-Baccalaureate Medicine, National Chung Hsing University, Taichung City 40227, Taiwan; 6 Research Center for Smart Sustainable Circular Economy, Tunghai University, No. 1727, Sec. 4, Taiwan Boulevard, Taichung City 407224, Taiwan; 7 Department of Medical Research, Kuang Tien General Hospital, Taichung City 43304, Taiwan

**Keywords:** tibial plateau fracture, deep learning, You Only Look Once, object detection, web-based system, clinical validation

## Abstract

**Background**: Tibial plateau fractures are complex knee injuries where timely and accurate diagnosis is critical to preventing long-term disability. In high-pressure emergency settings, the risk of missed fractures (false negatives) remains a significant challenge. **Objective**: This study aims to develop a robust, clinically safe automated detection model using advanced deep learning architectures. **Methods**: We utilized a dataset of 1489 real-world clinical X-ray images, annotated by orthopedic surgeons, to train and evaluate five versions of the You Only Look Once (YOLO) algorithm (v8, v9, v10, v11, and v12). A rigorous two-stage evaluation process was implemented. First, an initial screening excluded YOLOv8 and YOLOv10 due to critical detection failures (“background errors”), in which the models failed to detect any object in the target region. Second, a comprehensive performance analysis identified YOLOv11 as the optimal architecture. Based on these results, the YOLOv11 model was integrated into a user-friendly, web-based diagnostic system using the Python Flask framework. **Results**: The YOLOv11 model achieved the highest Mean Average Precision (mAP) of 99.3% and an Accuracy of 98.32%. Crucially for clinical safety, YOLOv11 demonstrated superior sensitivity (96.58%) with the lowest false negative rate, attributed to its enhanced feature aggregation capabilities which effectively distinguish subtle fracture lines from trabecular bone patterns. Independent web interface validation (n = 109 real-world cases) confirmed 98.17% accuracy, 98.00% sensitivity, 98.31% specificity, 98.00% Positive Predictive Value (PPV), and 98.31% Negative Predictive Value (NPV). **Conclusions**: This system is designed to support clinical workflows by providing real-time, highly accurate second opinions, thereby reducing diagnostic errors and alleviating radiologist workload.

## 1. Introduction

Tibial plateau fractures are among the most serious injuries involving the structures around the knee joint, as they disrupt the integrity of the articular surface [1]. Because the tibial plateau bears most of the mechanical load transmitted through the knee, damage to this region can result in severe pain, swelling, and subsequent impairment of mobility. If not accurately diagnosed and treated in a timely manner, these fractures may lead to nonunion, malunion, post-traumatic arthritis, and other chronic musculoskeletal disorders that significantly compromise lower-limb function and daily activities [2]. Early and precise identification of tibial plateau fractures is therefore essential for orthopedic clinical decision-making.

The emergency department is often the first point of contact for patients with fractures, where time-critical decision making and constraints on imaging and manpower make diagnostic efficiency and resource allocation particularly challenging [3]. In this high pressure setting, clinicians must rapidly interpret radiographs and determine management pathways, often under conditions of information overload and limited specialist support. When timely and accurate tools are lacking, inaccurate triage or imprecise initial assessments can lead to missed or delayed diagnoses, postponed definitive treatment, and ultimately poorer clinical outcomes. For injuries such as tibial plateau fractures, these delays may translate into prolonged hospitalization, more complex surgical procedures, and substantially higher healthcare utilization and costs [4]. Taken together, these challenges underscore the need for a convenient, easy to use diagnostic aid that can be seamlessly integrated into emergency workflows to support rapid, reliable fracture detection and reduce the cognitive burden on frontline clinicians.

In recent years, artificial intelligence (AI) has been increasingly applied to the diagnosis of skeletal disorders on X-ray images [5]. Previous studies have demonstrated the feasibility of AI-assisted tibial plateau fracture detection using diverse deep learning approaches, including conventional convolutional neural network backbones such as GoogleNet and ResNet, object detection frameworks such as RetinaNet, and earlier generations of You Only Look Once (YOLO) architectures [6,7,8,9]. However, a systematic head-to-head comparison of recent real-time object detection architectures for tibial plateau fracture detection under a consistent dataset and evaluation framework remains lacking. This represents the primary knowledge gap addressed in the present study. Such direct comparison is particularly important because advances across successive model generations may not necessarily translate into improved performance in detecting the subtle radiographic features of tibial plateau fractures. As a secondary implementation gap, most previous studies have focused primarily on algorithm development and offline performance evaluation, with limited investigation of translating a selected model into an accessible web-based clinical decision-support system followed by independent system-level validation. Accordingly, the present study systematically compares recent real-time object detection architectures under consistent experimental conditions, selects the most suitable model based on comparative performance, and subsequently integrates the selected model into a web-based diagnostic support system for further evaluation using an independent cohort of clinical cases.

Among the many object detection algorithms, YOLO is particularly relevant to the intended clinical application of this study because it combines object classification and spatial localization within a single-stage detection framework [10]. For tibial plateau fracture screening, this enables the system not only to identify the presence of a fracture but also to localize the suspected fracture region using a bounding box, providing interpretable spatial information to clinicians. Its computationally efficient single-stage design is also well suited to rapid image analysis and web-based clinical decision support. These characteristics motivated the selection of the YOLO family for the present study. With successive iterations, newer YOLO versions have introduced improvements in feature aggregation, multi-scale representation, and training strategies, providing better balances between accuracy and speed and enabling more robust detection of subtle findings compared with earlier generations [11,12]. Nevertheless, the performance of YOLO still varies across different versions and application domains. To address this, the present study compares several modern YOLO architectures, including YOLOv8, YOLOv9, YOLOv10, YOLOv11, and YOLOv12, in the specific task of detecting tibial plateau fractures on X-ray images. In addition, most previous works have focused solely on model-level performance metrics and retrospective evaluations, often without involving real-time clinical workflows [8]. This lack of an integrated validation environment makes it difficult to assess how such systems could be deployed in actual practice and how their outputs would influence decision-making at the point of care. The web-based diagnostic system developed in this study is designed to fill this gap by enabling side-by-side evaluation of AI predictions and expert interpretations, providing standardized test conditions, and facilitating practical adoption in routine clinical workflows.

The primary objective of this study is to systematically compare five recent YOLO architectures for tibial plateau fracture detection under consistent experimental conditions and identify the model with the most suitable diagnostic performance. Model performance is evaluated using object-detection and image-level diagnostic metrics, including mAP, accuracy, sensitivity, specificity, precision, and F1-score. The selected architecture is subsequently integrated into a prototype web-based clinical decision-support system and further evaluated using an independent cohort in a simulated clinical workflow.

## 2. Literature Review and Related Works

### 2.1. Deep Learning-Based Image Recognition

Image recognition stands as a core and extensively utilized domain within computer vision. It aims to equip machines with the ability to mimic human visual perception, enabling them to interpret and analyze visual content for tasks including classification, object detection, localization, and segmentation. As a core enabling technology, image recognition plays a vital role across numerous intelligent systems, particularly in fields such as smart healthcare, autonomous driving, surveillance, and industrial automation [13].

In contrast, deep learning offers a paradigm shift through the use of multi-layered neural network architectures that are especially well-suited for processing unstructured data such as images, speech, and natural language [14]. The remarkable progress in this field has been driven by three primary factors: the rapid expansion of available data, the increasing accessibility of high-performance computing resources such as graphics processing units (GPUs) and tensor processing units (TPUs), and ongoing innovations in neural network designs, including convolutional neural networks (CNNs), recurrent neural networks (RNNs), and Transformers. In standardized benchmark competitions such as ImageNet [15], deep learning models have reached or even surpassed the accuracy levels of human experts [16], facilitating the widespread implementation of artificial intelligence in diverse domains such as medical diagnostics, speech-enabled interfaces, and semantic information processing.

In recent years, the rapid progress in deep learning, particularly the widespread application of CNNs, has significantly transformed the field of image recognition [17]. CNNs are capable of performing end-to-end learning, automatically extracting hierarchical and abstract features from raw image data without relying on manually designed features [18]. This approach has led to substantial improvements in both recognition accuracy and model generalization. As a result, CNN-based techniques have been extensively deployed in practical applications such as facial recognition systems, perception modules for autonomous vehicles, automated industrial defect detection, and medical image interpretation [19].

### 2.2. Data Augmentation

Data augmentation is a commonly employed technique in the deep learning training process, aimed at generating new training samples by applying various random or systematic transformations to the original data. Its main purpose is to enhance the generalization capability of models and to reduce the risk of overfitting [20]. To enhance the model’s ability to learn robust feature representations, a variety of data augmentation techniques are typically employed. These methods, which include image rotation, flipping (horizontally and vertically), random cropping, scaling, and the addition of Gaussian noise, also involve adjusting parameters like brightness, contrast, and color distribution. Other common transformations are blur and affine transformations, all of which serve to significantly increase dataset diversity.

In fields such as medical image analysis and industrial inspection, data acquisition is often limited by high costs, ethical constraints, or sample scarcity. Thus, data augmentation holds particularly critical value in these areas. Properly applied data augmentation not only compensates for the limitations of small datasets but also improves the accuracy and stability of models, thereby enhancing their practical performance. As such, data augmentation is regarded as one of the key techniques for improving model performance, playing an indispensable role, especially in small-sample learning scenarios.

### 2.3. Convolutional Neural Network (CNN)

CNNs are a type of neural network that extracts features hierarchically. Drawing inspiration from the human visual system, they first identify local patterns and then build upon these to recognize global, semantic features. This layered feature extraction process makes CNNs the predominant architecture for analyzing two-dimensional image data. These architectural advantages have enabled CNNs to achieve exceptional performance across a wide range of tasks, including image classification, speech recognition, and medical image analysis. In particular, CNNs have demonstrated high accuracy in applications such as tumor detection, pneumonia screening, and the identification of bone fractures [21].

### 2.4. You Only Look Once (YOLO)

The YOLO family of algorithms is a single-stage object detection framework built on CNNs. Its key feature is the simultaneous execution of object classification and bounding box regression in a single network pass, a method that substantially decreases inference latency when compared to two-stage detectors like Region-based Convolutional Neural Networks (R-CNN) and Faster R-CNN [22]. Through successive iterations, including YOLOv3, YOLOv4, YOLOv5, and the most recent YOLOv12, the YOLO series has progressively optimized the trade-off between detection accuracy, computational speed, and model compactness. These enhancements have enabled widespread adoption in medical imaging and smart healthcare applications systems [23].

The selection of YOLO for the present study was motivated by its suitability for the intended clinical application. Unlike pure image-classification approaches, object detection provides both fracture identification and spatial localization, allowing the suspected fracture region to be visualized for clinicians. Although alternative detectors such as Faster R-CNN, RetinaNet, DETR, and RT-DETR may also be applicable, YOLO combines localization and classification within a computationally efficient single-stage framework, making it well suited to rapid, web-based clinical decision support.

In the field of intelligent healthcare, YOLO-based models have been increasingly incorporated into various diagnostic imaging tasks. Previous studies have validated the utility of YOLO in assisting with the detection of proximal femoral fractures and spinal fractures, demonstrating improvements in diagnostic speed and a reduction in the risk of misdiagnosis [24,25]. YOLO’s efficient feature extraction and low-latency inference make it particularly suitable for time-sensitive environments, such as emergency care or preliminary triage, where it can serve as a valuable decision-support tool for clinicians.

Despite these advancements, the application of YOLO in the automated detection of tibial plateau fractures remains relatively underexplored. According to Huo et al., YOLO-based deep learning models have been successfully applied to adult tibial plateau fracture diagnosis and demonstrated promising performance compared with radiologists; however, their work did not systematically compare different YOLO versions or examine architecture-specific effects on detection accuracy [7]. In addition, Wang et al. investigated the impact of different radiographic views and earlier YOLO architectures on fracture detection and highlighted the benefits of multi-view modeling. Nevertheless, that study did not incorporate the latest YOLO versions and did not implement a web-based system for real-time clinical testing, leaving a gap between algorithm development and practical deployment in routine workflows [8]. Given the subtle, heterogeneous nature of tibial plateau fractures in radiographic imaging, this diagnostic task presents considerable challenges for object detection models. It demands high precision in feature extraction and interpretation of fine-grained anatomical details. Therefore, a systematic comparison of various YOLO versions for this particular application could offer critical insights into optimizing AI-assisted diagnostic tools in orthopedic imaging.

To address this research gap, the present study adopts a structured modeling and evaluation strategy for tibial plateau fracture detection. Specifically, we train and compare recent YOLO architectures, including YOLOv9, YOLOv11, and YOLOv12, using an annotated clinical X-ray dataset, and integrate the best performing model into a web-based diagnostic support system with real-time feedback and a user-friendly interface. The overarching objective is to enhance diagnostic efficiency, reduce the workload on healthcare providers, and facilitate the practical implementation of AI-enabled tools in orthopedic emergency care. Given YOLO’s unified architecture and real-time detection capabilities, its role in medical imaging is likely to become increasingly important. As these models continue to improve in precision and robustness, they are expected to serve as a core component of intelligent clinical decision support systems, enabling more accurate, efficient, and scalable diagnostic workflows.

## 3. Methods

### 3.1. System Architecture

This study aims to develop a deep learning-based model capable of automatically detecting tibial plateau fractures, integrated within a web-based interface to create a system that emphasizes both practical usability and operational convenience. The overall system architecture and the research workflow are outlined as follows.

To ensure system stability and optimal performance, the development environment was configured on an Ubuntu 22.04 operating system, equipped with an NVIDIA Geforce RTX 4080 GPU, NVIDIA Corporation, Santa Clara, CA, USA. This hardware setup significantly accelerated the training and inference processes of the YOLO models. The complete system architecture is illustrated in Figure 1.

To clearly illustrate the data processing workflow and model construction steps, Figure 2 schematically represents the complete process from raw image segmentation and augmentation to model training and prediction. First, knee X-ray images were preprocessed and then partitioned into training, validation, and test sets with a distribution of 70%, 10%, and 20%, respectively. The validation set was used for hyperparameter tuning and monitoring model performance, whereas the test set was used for evaluation after all model architectures and training configurations had been finalized. To improve the model’s generalization capabilities, the training set was subsequently expanded using various data augmentation techniques. Subsequently, several versions of the YOLO object detection algorithm were thoroughly trained to construct a robust fracture detection model. To maintain fairness in the evaluation process, an initial screening was conducted on the test set to identify models exhibiting critical detection failures (background errors), defined as failure to detect any object in the target region. Models with such failures were excluded, while those with zero background errors were included in the subsequent performance comparison and detailed analysis. No model retraining or hyperparameter adjustment was performed based on the test-set results. The final performance evaluation was conducted using the test data, and the model demonstrating the best overall performance was selected for integration into the final web-based diagnostic system, which offers a user-friendly interface designed to facilitate real-time interaction and efficient clinical deployment. Following model selection and integration, the web-based diagnostic system underwent rigorous testing through the Flask interface to validate real-time inference performance and clinical usability.

### 3.2. Data Acquisition

For data acquisition, knee X-ray images, including both anteroposterior (AP) and lateral views, were obtained from a tertiary referral center. Both fracture and non-fracture cases were included, while images containing implants, old fractures, or fractures in locations other than the tibial plateau were excluded to ensure data quality and consistency. After applying these inclusion and exclusion criteria, a total of 1489 X-ray images, comprising 746 AP and 743 lateral views, were retained for model training and offline evaluation, as detailed in Table 1. Additionally, an independent retrospective cohort of 109 patients (109 knee radiographs; 50 fracture and 59 non-fracture) was collected from the same tertiary referral center using the same inclusion and exclusion criteria as the original dataset. Each patient contributed one radiograph, and these 109 radiographs were entirely independent of the 1489 images used for model development and internal evaluation. This cohort was used specifically for diagnostic validation of the deployed Flask-based system under the same Institutional Review Board approval as the original study. Prior to classification and annotation, all images underwent preprocessing procedures including resizing, contrast enhancement, and noise reduction to ensure high-quality input for model training. To preserve the original anatomical aspect ratio and avoid anisotropic distortion of fine fracture-related features, the high-resolution radiographs were proportionally resized to fit within the 640 × 640 model input, with letterbox padding applied to the remaining regions. No patch-based cropping was performed. To maintain the clinical accuracy of the dataset, annotations were carefully reviewed and verified by two experienced orthopedic physicians. This process ensured consistency across the dataset and improved the overall reliability of the model.

### 3.3. Data Classification and Annotation

All images were independently screened and classified by two experienced orthopedic physicians based on the presence or absence of fractures, with CT scans used as the reference standard to assist in confirming the diagnosis when available. Each image was subsequently classified as either “fracture” or “non-fracture”. The image marking operation was performed using the Roboflow platform to obtain the bounding box, which allows the machine to train the eigenvalues for specific locations to improve the accuracy and is limited to the tibial plateau area [26,27]. For fracture images, the ground-truth bounding box was defined to enclose the fracture region of the tibial plateau, including the visible fracture and its immediately adjacent involved area, rather than the fracture line alone or the entire tibial plateau. Non-fracture images contained no fracture bounding boxes. Figure 3 illustrates examples of the annotated features. This rigorous data classification and annotation process ensured that the models were trained with high-quality and medically accurate data.

### 3.4. Data Augmentation

Due to the limited size of medical imaging datasets, directly training models on the original data could lead to reduced accuracy and overfitting. To address this issue, this study utilized the Roboflow platform to implement a robust data augmentation strategy to increase dataset diversity and enhance model generalization. The applied augmentation techniques included image cropping; horizontal and vertical flipping; adjustments of brightness, exposure, and contrast; random rotation; and noise injection. By employing these various transformations, the dataset was expanded to increase variability. Following augmentation, the volume of the tibial plateau X-ray dataset significantly increased, as shown in Table 1. These additional samples enabled the models to learn more varied patterns, effectively mitigating the risk of overfitting and improving their adaptability and robustness for real-world clinical applications across a wider array of clinical scenarios.

### 3.5. Model Training

To ensure fairness and consistency in the comparison, all models were trained under identical hyperparameter settings, including a batch size of 8 images per update, an input image size of 640 × 640 pixels, and 500 training epochs. Furthermore, the dataset was partitioned at the image level into three subsets, with 70% allocated for training, 10% for validation, and 20% for testing. The training set was used to optimize the model parameters, while the validation set was used to fine-tune hyperparameters and monitor training to prevent overfitting. All model architectures and training configurations were finalized before evaluation on the test set, and no model retraining or hyperparameter adjustment was performed based on the test-set results. Through a consistent and rigorous training process, the models were trained under identical environments, ensuring objectivity and reproducibility of the comparative results.

### 3.6. Loss Function Formulation

To optimize the model parameters for tibial plateau fracture detection, the total loss function $L_{total}$ is composed of three distinct components: classification loss, bounding box regression loss, and distribution focal loss.

#### 3.6.1. Classification Loss

The classification loss ($L_{cls}$) utilizes Binary Cross-Entropy (BCE) to minimize the error in fracture probability prediction. It is defined as

(1) $$L_{cls}=-\frac{1}{N}\sum_{i=1}^{N}\left[y_{i}\log\left({\hat{p}}_{i}\right)+\left(1-y_{i}\right)\log\left(1-{\hat{p}}_{i}\right)\right]$$

where *N* represents the number of samples, $y_{i}$ denotes the ground truth label (1 for fracture, 0 for non-fracture), and ${\hat{p}}_{i}$ is the predicted probability.

#### 3.6.2. Bounding Box Regression Loss

To ensure precise localization of the fracture line, we employ the Complete Intersection over Union (CIoU) loss. This metric considers overlap area, center point distance, and aspect ratio:

(2) $$L_{box}=1-IoU+\frac{\rho^{2}\left(b,b^{gt}\right)}{c^{2}}+\alpha v$$

where IoU is the intersection over union between the predicted box b and the ground truth box $b^{gt}$. ρ(·) represents the Euclidean distance between the center points of the two boxes, and *c* is the diagonal length of the smallest enclosing box covering both. The terms α and *v* are regularization parameters that enforce aspect ratio consistency.

#### 3.6.3. Distribution Focal Loss

To handle the ambiguity of fracture boundaries in X-ray images, Distribution Focal Loss (DFL) is used to refine the bounding box edges by modeling them as a general distribution:

(3) $$L_{DFL}\left(S_{i},S_{i+1}\right)=-\left(\left(y_{i+1}-y\right)\log\left(S_{i}\right)+\left(y-y_{i}\right)\log\left(S_{i+1}\right)\right)$$

where *y* is the continuous target label, and $y_{i},y_{i+1}$ are the nearest integer values ($y_{i}\leq y\leq y_{i+1}$). $S_{i}$ and $S_{i+1}$ represent the predicted probabilities at these locations. This allows the network to focus on the probabilistic distribution of the fracture boundaries rather than a single deterministic coordinate.

### 3.7. Performance Evaluation Metrics and Statistical Analysis

To rigorously evaluate model performance, standard object-detection and binary diagnostic metrics were employed. For object-detection performance, mean Average Precision (mAP) was evaluated at an IoU threshold of 0.50 (mAP@0.5) and averaged across IoU thresholds from 0.50 to 0.95 in increments of 0.05 (mAP@0.5:0.95).

For image-level diagnostic evaluation, a confidence threshold of τ=0.50 was applied to determine the presence or absence of a fracture prediction. After Non-Maximum Suppression (NMS) with an IoU threshold of 0.45, an image was classified as fracture-positive if at least one fracture bounding box with a confidence score ≥0.50 remained; otherwise, it was classified as fracture-negative. Image-level diagnostic classifications were defined as follows:

- True Positive (TP): A fracture image classified as fracture-positive.
- False Positive (FP): A non-fracture image classified as fracture-positive.
- False Negative (FN): A fracture image classified as fracture-negative.
- True Negative (TN): A non-fracture image classified as fracture-negative.

Diagnostic performance was quantified using accuracy, sensitivity (recall), specificity, precision (Positive Predictive Value, PPV), Negative Predictive Value (NPV), and F1-score:

(4) $$Accuracy=\frac{TP+TN}{TP+TN+FP+FN}$$

(5) $$Sensitivity\;\left(Recall\right)=\frac{TP}{TP+FN}$$

(6) $$Specificity=\frac{TN}{TN+FP}$$

(7) $$Precision\;\left(PPV\right)=\frac{TP}{TP+FP}$$

(8) $$NPV=\frac{TN}{TN+FN}$$

(9) $$F1\text{-}score=2\cdot \frac{Precision\cdot Recall}{Precision+Recall}$$

Two-sided 95% confidence intervals (CIs) for the binomial diagnostic proportions were computed using the Wilson score method.

### 3.8. Model Selection and Initial Screening

To identify the most viable architectures for this specific medical task, we conducted a preliminary screening of five YOLO versions: YOLOv8, YOLOv9, YOLOv10, YOLOv11, and YOLOv12. Each model was trained under identical hyperparameters to ensure a fair comparison.

The primary exclusion criterion was clinical safety, with particular emphasis on missed fractures, as FNs are substantially more harmful than FPs in medical diagnosis. A conventional FN was defined as a case in which the target region was detected but incorrectly classified as non-fracture, whereas a background error was defined as a critical detection failure in which no object was detected in the target region. Although both result in missed diagnoses, a background error was considered more critical because the model provides neither a bounding box nor a visual warning, essentially becoming “blind” to the target region. Such failures were therefore considered critical detection errors in the present analysis.

As illustrated in Figure 4, YOLOv8 exhibited one background error in addition to eight conventional FNs, whereas YOLOv10 exhibited two background errors in addition to seven conventional FNs. Consequently, both models were excluded. In contrast, YOLOv9, YOLOv11, and YOLOv12 demonstrated zero background errors and were retained for further comparative evaluation. The results of the initial screening and final model selection are summarized in Table 2.

## 4. Results

### 4.1. Training Stability and Convergence

To assess the learning efficiency and stability of the selected models, we analyzed the training dynamics of YOLOv9, YOLOv11, and YOLOv12 based on their loss curves (Figure 5). The training loss curves for all three models demonstrate a consistent downward trajectory, indicating effective convergence. However, a comparative analysis reveals distinct differences in stability. YOLOv9 exhibits a diverging trend between training and validation loss in the final 100 epochs, indicating a tendency toward overfitting on the training data. The validation loss for YOLOv11 remains closely aligned with the training loss throughout the process, demonstrating the tightest synchronization and a smooth, monotonic validation curve, showing minimal divergence as its feature extraction (via the C3k2 block) learns robust anatomical features rather than memorizing noise [28]. In contrast, YOLOv12 shows minor oscillations in the validation box loss during the mid-training phase, suggesting that its complex architecture struggled slightly more to converge on the bounding box regression task. These results indicate that YOLOv11 achieved the most stable learning process among the evaluated models.

### 4.2. Comparative Detection Performance

Following training, the models were evaluated on the independent test set. The comparative performance is detailed below using confusion matrices and metric curves to assess differences in detection performance across the selected YOLO models.

#### 4.2.1. Classification Accuracy (Confusion Matrix Analysis)

To visualize the specific classification behaviors, the confusion matrices are presented in Figure 6. A critical comparison of FN shows that YOLOv11 missed 5 fractures, whereas the other versions each missed 7.

#### 4.2.2. Sensitivity and Model Confidence

The Recall–Confidence curves (Figure 7) illustrate how model sensitivity changes as the confidence threshold increases. As observed, YOLOv11 maintains a stable recall rate (>0.9) even at higher confidence thresholds compared to YOLOv12. This indicates that YOLOv11 is “more sure” of its correct fracture predictions, whereas YOLOv12 requires a lower confidence threshold to achieve the same sensitivity, potentially introducing more noise.

#### 4.2.3. F1-Score Stability

The F1-score curves (Figure 8) provide a composite view of precision and recall. YOLOv11 exhibits the most robust F1 curve with a broad, flat plateau. This indicates that the model’s performance remained relatively stable across a range of confidence thresholds.

#### 4.2.4. Precision–Recall Trade-Off

The Precision–Recall curves (Figure 9) demonstrate consistently high detection performance across all three models. YOLOv11 and YOLOv12 achieved the highest mAP@0.5 of 99.3%, slightly exceeding the 99.1% achieved by YOLOv9. The curves remained close to the upper-right region across a wide recall range, indicating a favorable balance between precision and recall.

#### 4.2.5. Model Performance Comparison

Table 3 summarizes the final numerical comparison of the three selected models. YOLOv11 achieved the highest accuracy (98.32%), sensitivity (96.58%), and F1-score (98.25%), while maintaining 100.00% specificity and precision. YOLOv9 also achieved 100.00% specificity and precision but showed lower accuracy (97.65%), sensitivity (95.21%), and F1-score (97.56%). YOLOv12 achieved an accuracy of 97.32%, a sensitivity of 95.21%, a specificity of 99.34%, precision of 99.29%, and an F1-score of 97.20%. Overall, YOLOv11 demonstrated the most balanced performance across the evaluated metrics.

#### 4.2.6. Clinical Safety Assessment: Missed Diagnosis Rate

To quantify the clinical risk associated with each model, we calculated the Missed Diagnosis Rate (MDR), equivalent to the false negative rate, using the following formula:

(10) $$MDR=\frac{FN}{TP+FN}\times 100\%$$

Based on the confusion matrix data presented in Section 4.2, YOLOv9 and YOLOv12 both incurred 7 missed fractures out of 146 actual positive cases, resulting in an MDR of 4.79%, while YOLOv11 missed only 5 fractures, achieving a reduced MDR of 3.42%. This represents a 28.6% relative reduction in clinical risk compared to the other architectures.

### 4.3. Web Design and System Implementation

Based on the comprehensive evaluation, YOLOv11 was selected as the core model for the proposed clinical support system. To enhance practical usability, a graphical web-based interface was developed using the Python (version 3.10) Flask framework.

#### 4.3.1. System Architecture and Usage

The system supports the uploading of common medical image formats (JPG, PNG, DICOM). As shown in Figure 10, the interface allows users to efficiently select images, initiate detection, and review results.

The system provides immediate visual feedback by rendering bounding boxes over detected fracture regions (Figure 11). Crucially, it includes a modular backend (Figure 12) that allows for seamless model updates, enabling the future integration of newer YOLO versions without altering the frontend workflow.

To support clinical documentation, the system includes features for downloading annotated images (Figure 13) and reviewing historical case logs (Figure 14), ensuring that the AI tool integrates smoothly into existing hospital data management workflows.

#### 4.3.2. Web Interface Testing Results

Following web system deployment, the selected model was rigorously tested through the Flask interface using an independent cohort of 109 real-world knee X-ray cases. The testing protocol simulated clinical workflow: images were uploaded via the web interface and processed in real time, and predictions were compared against expert ground truth. The web interface validation results are summarized in Table 4.

The web-deployed YOLOv11 system achieved an accuracy of 98.17% (95% CI: 93.56–99.50%), a sensitivity of 98.00% (95% CI: 89.50–99.65%), a specificity of 98.31% (95% CI: 91.00–99.70%), a PPV of 98.00% (95% CI: 89.50–99.65%), and an NPV of 98.31% (95% CI: 91.00–99.70%), with only two discordant cases (1 FN, 1 FP). Both errors occurred at low confidence scores (<0.85), which were automatically flagged for physician review. These results demonstrate the retrospective diagnostic performance of the web-based system in the independent validation cohort.

## 5. Discussion

This study systematically evaluated five recent YOLO architectures for tibial plateau fracture detection and further translated the selected model into a web-based clinical support system. The initial screening revealed critical detection failures (“background errors”) in YOLOv8 and YOLOv10, leading to their exclusion from subsequent comparison. Among the remaining models, YOLOv11 demonstrated the most balanced overall performance, achieving the highest accuracy (98.32%), sensitivity (96.58%), and F1-score (98.25%), while producing the fewest false negatives. Following deployment, independent web interface validation using real-world cases confirmed that the system maintained high diagnostic performance.

Previous studies have also demonstrated the feasibility of deep learning for tibial plateau fracture detection. Liu et al. reported an accuracy of 0.91 for AI-based tibial plateau fracture detection, comparable to the performance of orthopedic physicians (0.92 ± 0.03) [6]. Huo et al. subsequently demonstrated the feasibility of deep learning for adult tibial plateau fracture diagnosis in a multicenter study with external validation [7]. Wang et al. further compared earlier YOLO architectures across different radiographic views and demonstrated the potential benefit of multi-view fracture detection [8]. More recently, Van der Gaast et al. investigated deep learning for both tibial plateau fracture detection and classification [9]. In comparison, the present study achieved a sensitivity of 96.58% (95% CI: 92.23–98.53%) and an mAP@0.5 of 99.3% with YOLOv11. However, direct numerical comparison across studies should be interpreted cautiously because of differences in datasets, study populations, model architectures, and evaluation metrics. The principal contribution of the present study is therefore not simply a higher numerical performance, but the systematic head-to-head evaluation of recent YOLO architectures under the same experimental framework, followed by independent validation of the selected model through a web-based system.

The findings also indicate that newer model versions do not necessarily provide superior performance for a specific medical imaging task. Although YOLOv12 represents a more recent architecture, YOLOv11 demonstrated greater training stability and better overall detection performance for tibial plateau fractures. One possible explanation is the compatibility between the model architecture and the radiographic characteristics of tibial plateau fractures. These fractures may present with subtle radiographic features that can be obscured by complex trabecular patterns, which may act as background noise during feature extraction. The C3k2-based architecture of YOLOv11 enhances feature extraction and aggregation efficiency, which may facilitate the preservation of subtle fracture-related features while reducing interference from background noise [28]. In contrast, the greater architectural complexity of YOLOv12 may have increased its sensitivity to background noise in this specific task, without providing a corresponding improvement in sensitivity.

A key contribution of this study is the incorporation of critical detection failure analysis into the model selection process. Rather than selecting the optimal architecture solely on the basis of aggregate performance metrics, YOLOv8 and YOLOv10 were excluded because of background errors (Figure 4). In medical diagnostics, false negatives are of greater clinical concern than false positives because missed abnormalities may delay further evaluation and treatment. More importantly, a background error represents a critical detection failure in which the model provides neither a bounding box nor a visual warning, essentially becoming “blind” to the target region. In a high-throughput emergency department, such a silent failure could result in a missed fracture and delayed treatment, potentially contributing to complications such as post-traumatic osteoarthritis or malunion. By explicitly screening for these critical detection failures, this study adopts a clinically oriented approach to model selection that considers failure patterns in addition to conventional performance metrics. Among the 146 fracture-positive test images, YOLOv11 missed 5 fractures (3.42%), compared with 7 fractures (4.79%) for both YOLOv9 and YOLOv12. No background errors were observed for YOLOv11 in the test cohort. The selection of YOLOv11 was therefore based not only on its high mAP (99.3%) but also on its observed failure pattern and lower false-negative rate in the present test cohort.

Another important contribution of this study is addressing the practical “last-mile” challenge of translating medical AI from algorithm development to clinical application. The selected YOLOv11 model was integrated into a lightweight Flask-based web system that provides real-time prediction and visual feedback through a standard web browser. Its modular backend (Figure 12) allows future integration of state-of-the-art detection models without substantial modification of the frontend workflow, enabling the system to evolve as newer architectures become available. Importantly, independent validation using 109 real-world cases demonstrated that the deployed system maintained high diagnostic performance, supporting its feasibility beyond offline model evaluation. This browser-based architecture may reduce dependence on specialized workstations and lower technical barriers to implementation, particularly in resource-constrained settings where extensive upgrades to existing hospital infrastructure or picture archiving and communication system (PACS) may be difficult. Overall, the proposed system provides a practical framework for translating AI-assisted fracture detection into clinical decision support, particularly in time-sensitive settings such as emergency care.

Several limitations should be acknowledged. First, this retrospective single-center study may limit the generalizability of the findings. Future studies should conduct multicenter external and prospective validation using larger and more diverse datasets. Second, the dataset was partitioned at the image level rather than at the patient level, and AP and lateral radiographs from the same patient may therefore have been assigned to different subsets. Although each radiograph was processed independently without patient identifiers or paired-view feature sharing, potential patient-level data leakage cannot be completely excluded. Future studies should adopt patient-level partitioning to ensure strict independence among the training, validation, and test cohorts. Third, the current system focuses on binary fracture detection using plain radiographs and does not assess different fracture morphologies or severity levels. Although binary fracture detection is valuable for rapid screening and triage, formal fracture classification is important for subsequent orthopedic treatment and surgical planning. In addition, the dataset was not specifically stratified according to fracture displacement or subtle/occult fracture status; therefore, further subgroup-specific evaluation is needed to determine model performance in subtle, occult, or nondisplaced fractures. Future studies should therefore extend the current framework to automated fracture classification and severity grading, including the Schatzker and AO/OTA classification systems. The integration of CT imaging and three-dimensional fracture characterization may further enable more comprehensive assessment and support surgical planning. Fourth, radiographs containing metallic implants or prior fractures were excluded from the current dataset, and model performance has therefore not been specifically validated in these conditions. Severe osteoarthritic changes and marked joint-space narrowing may also affect model predictions. The current web interface does not include dedicated automated screening for these unsupported image characteristics; therefore, predictions in such cases should be interpreted cautiously and require physician review. Future development should incorporate automated image-quality and out-of-distribution screening to flag such cases before AI-assisted interpretation. Fifth, although no model retraining or hyperparameter adjustment was performed after test-set evaluation, test-set findings were used to exclude models with background errors from the subsequent detailed comparison, which may introduce selection bias. In addition, the clinical significance and generalizability of background-error screening have not yet been independently validated. Future studies should perform model screening exclusively on the validation set, reserve an independent test set for final performance evaluation, and further validate the value of background errors as a model-selection criterion. Finally, the independent web validation assessed system-level diagnostic performance rather than its direct impact on clinical decision-making. Prospective studies comparing physicians with and without AI assistance are needed to evaluate effects on missed diagnoses, interpretation time, and diagnostic consistency. Future integration with hospital PACS should also be explored.

## 6. Conclusions

This study systematically compared multiple YOLO architectures for tibial plateau fracture detection and identified YOLOv11 as the most balanced model, with high diagnostic performance, zero background errors, and the lowest false negative rate. Its successful integration into a web-based system and independent validation further demonstrated the feasibility of translating the model into clinical decision support.

## Figures and Tables

**Figure 1:**
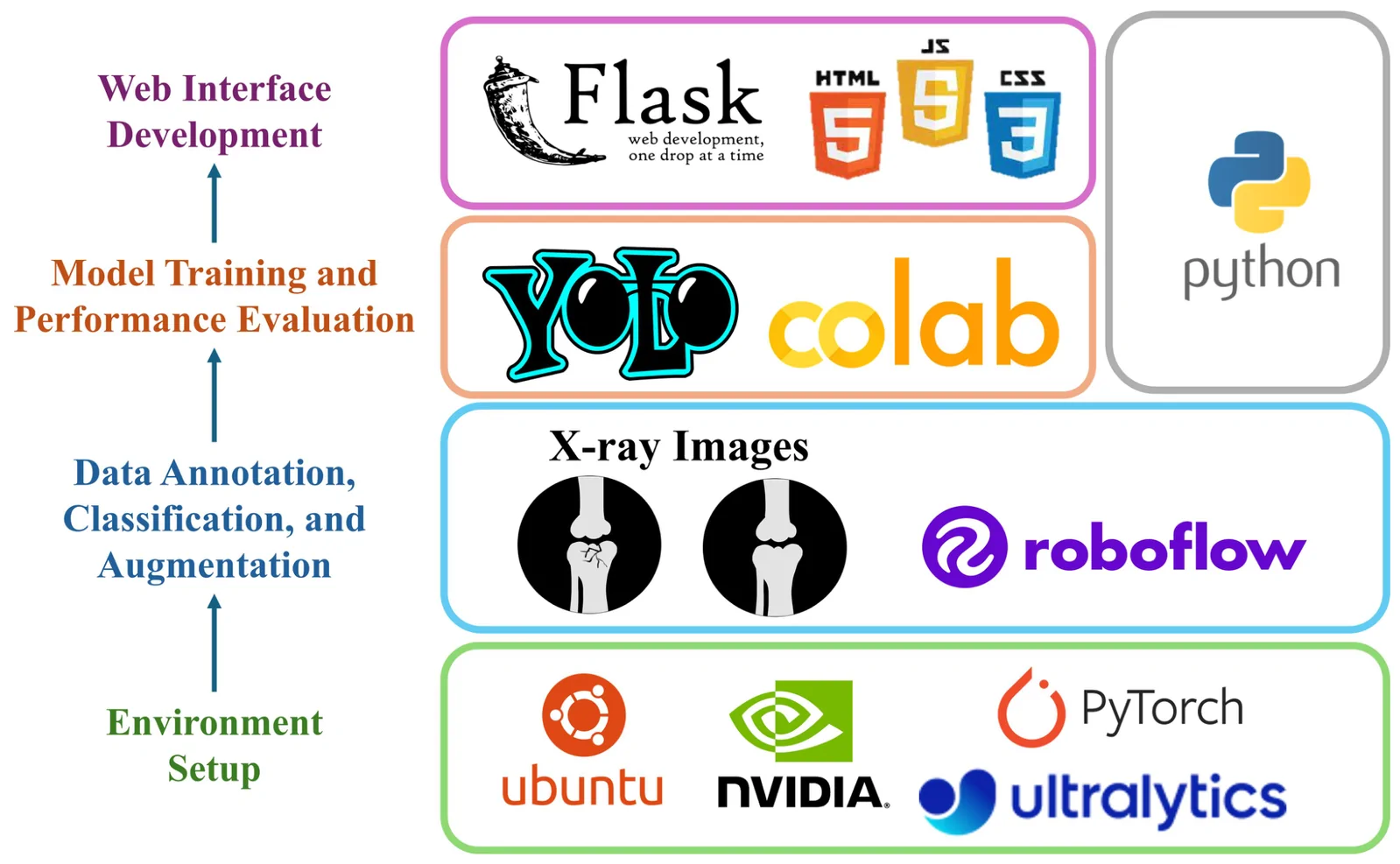
System architecture. Pipeline includes environment setup, X-ray data annotation, YOLO model training in Colab, and deployment via a Flask web interface.

**Figure 2:**
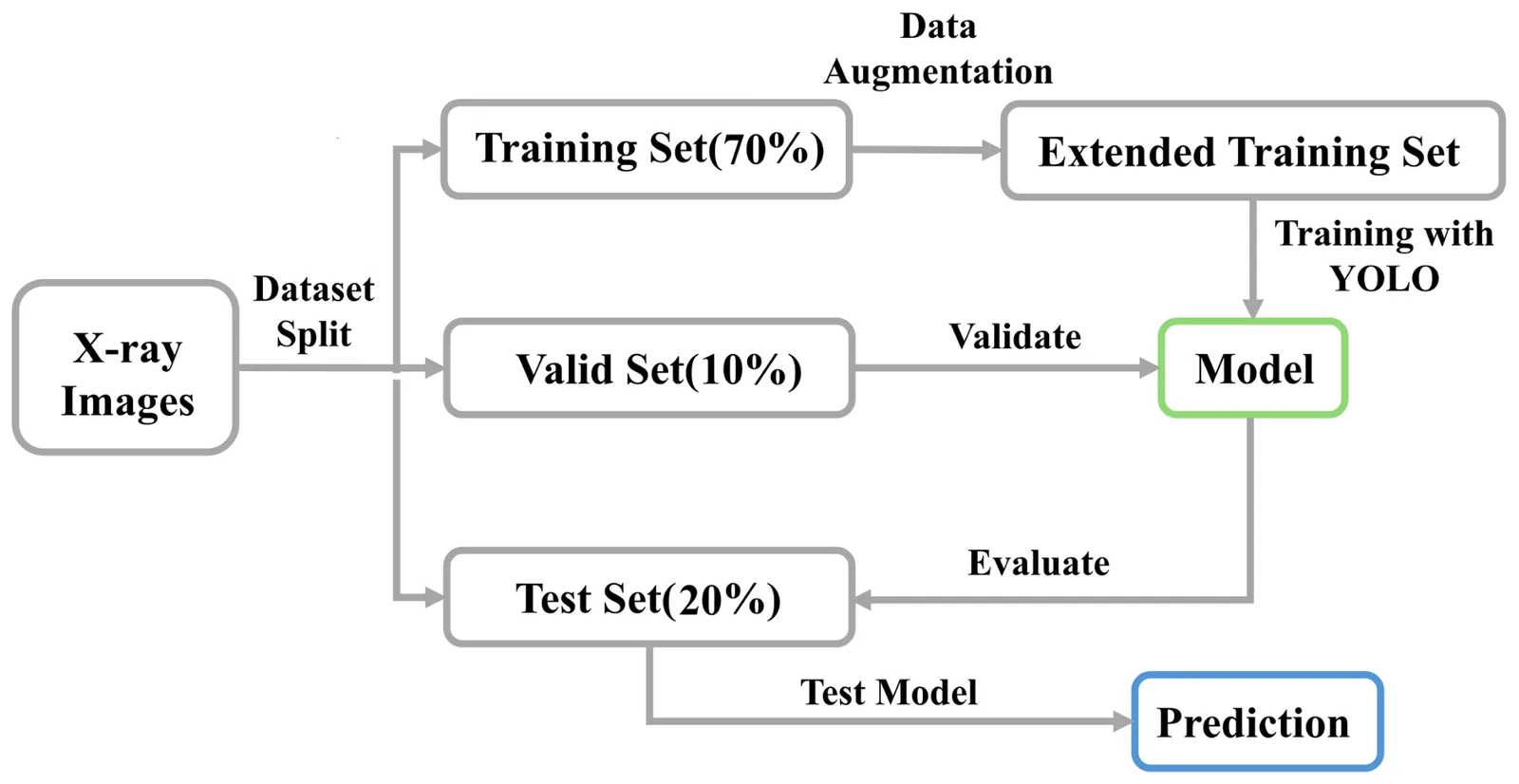
Data processing. X-ray images were split into training (70%), validation (10%), and test (20%) sets. The training set was augmented for YOLO model training, with validation and test sets used for evaluation and prediction.

**Figure 3:**
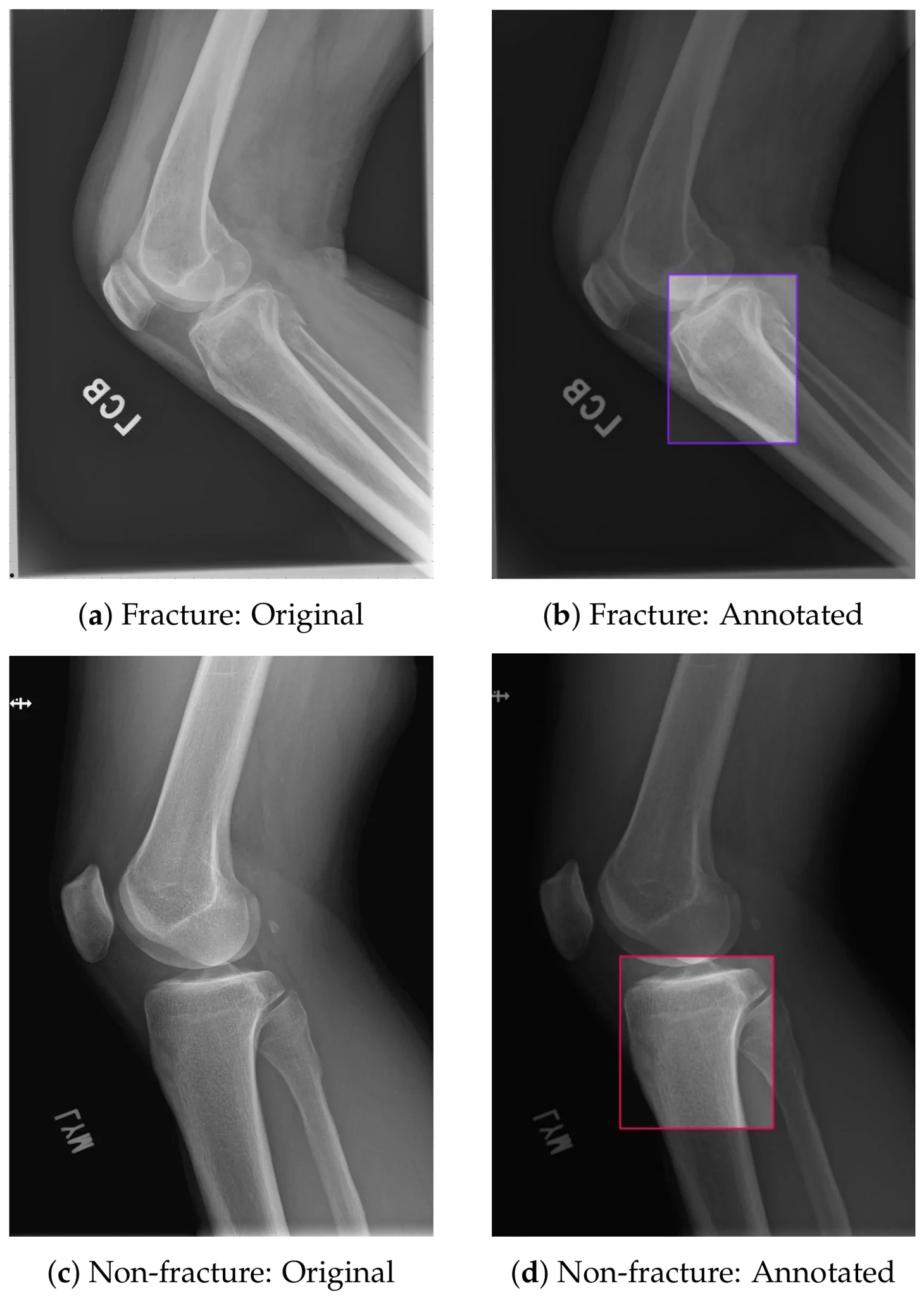
Data annotation. Examples of fracture and non-fracture X-ray images with corresponding bounding box annotations.

**Figure 4:**
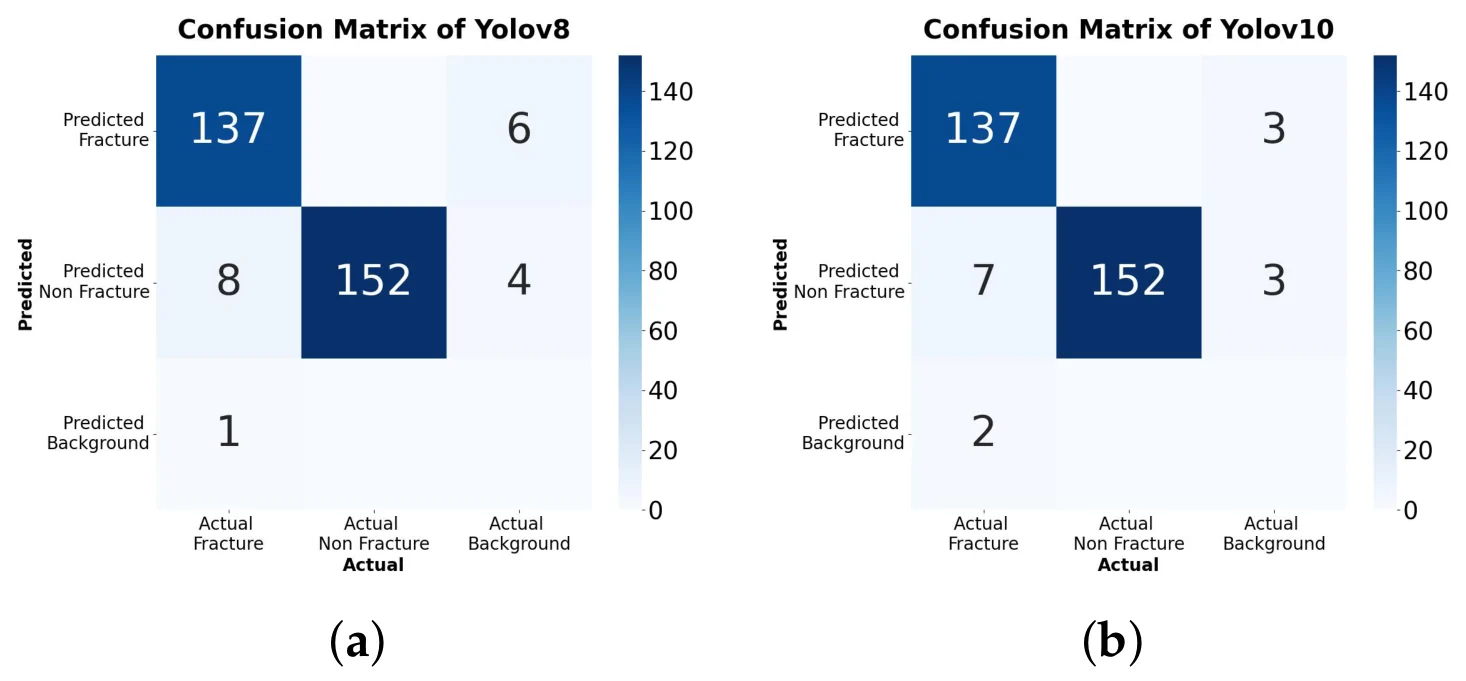
Confusion Matrix Anomalies detected during screening. The matrices highlight instances where the models failed to detect any object (Fracture classified as Background), rendering them clinically unsafe. (**a**) Critical Background Error in YOLOv8. (**b**) Critical Background Error in YOLOv10.

**Figure 5:**
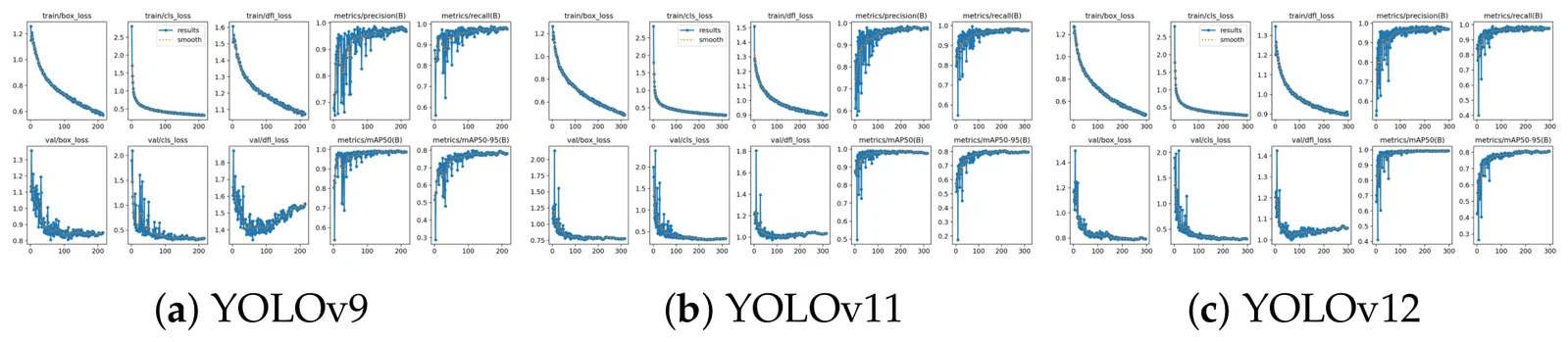
Training performance comparison across YOLO models. Training loss and performance metrics for YOLOv9, YOLOv11, and YOLOv12.

**Figure 6:**
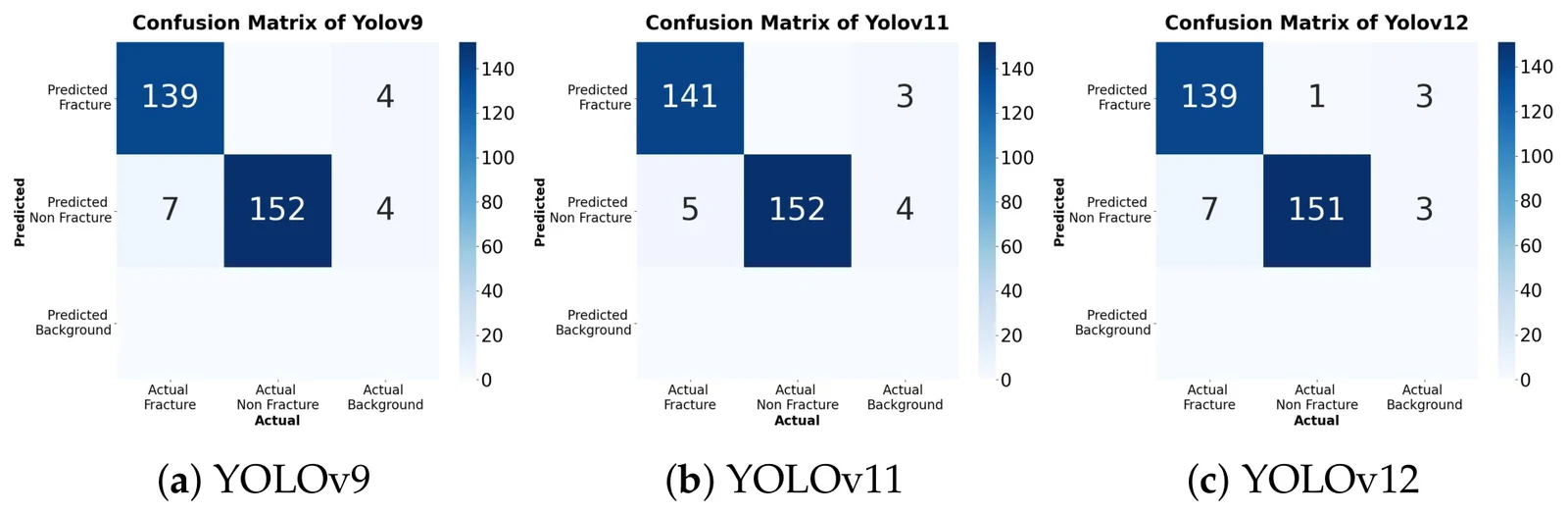
Comparative Confusion Matrices. YOLOv11 (**b**) demonstrates the lowest number of False Negatives (5), compared to 7 for both YOLOv9 and YOLOv12.

**Figure 7:**
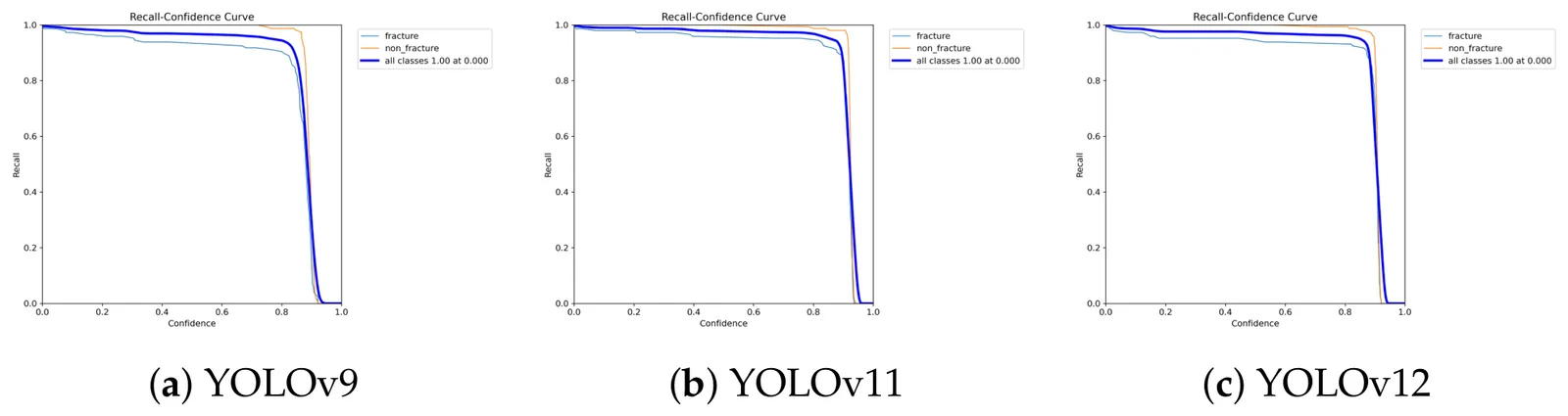
Recall–Confidence Curves. YOLOv11 maintains near-perfect recall (top-right plateau) for a wider range of confidence scores compared to YOLOv12, indicating higher model certainty for fracture cases.

**Figure 8:**
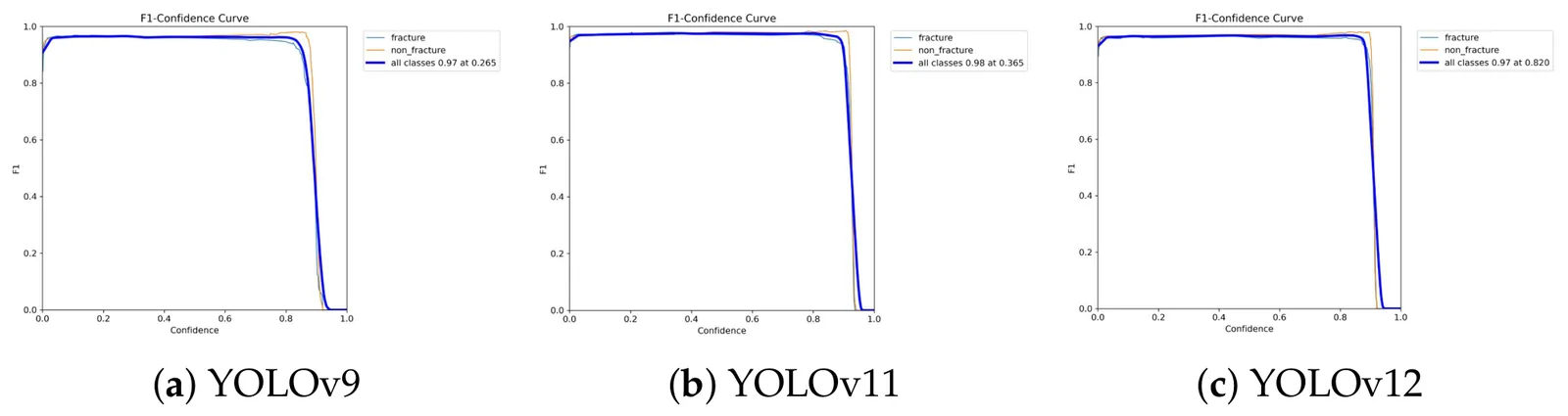
F1-Score Curves. The plateau of the YOLOv11 curve is flatter and more extended than YOLOv9, reflecting superior stability across varied thresholds.

**Figure 9:**
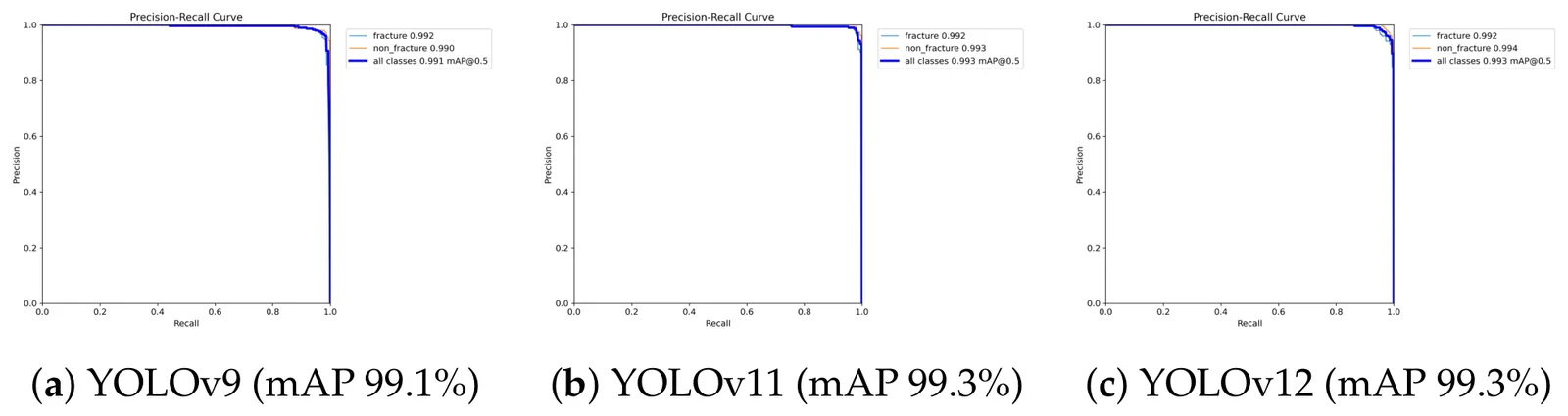
Precision–Recall Curves. While all models perform well, YOLOv11 achieves the optimal balance (mAP 99.3%) without the slight trade-off in specificity observed in YOLOv12.

**Figure 10:**
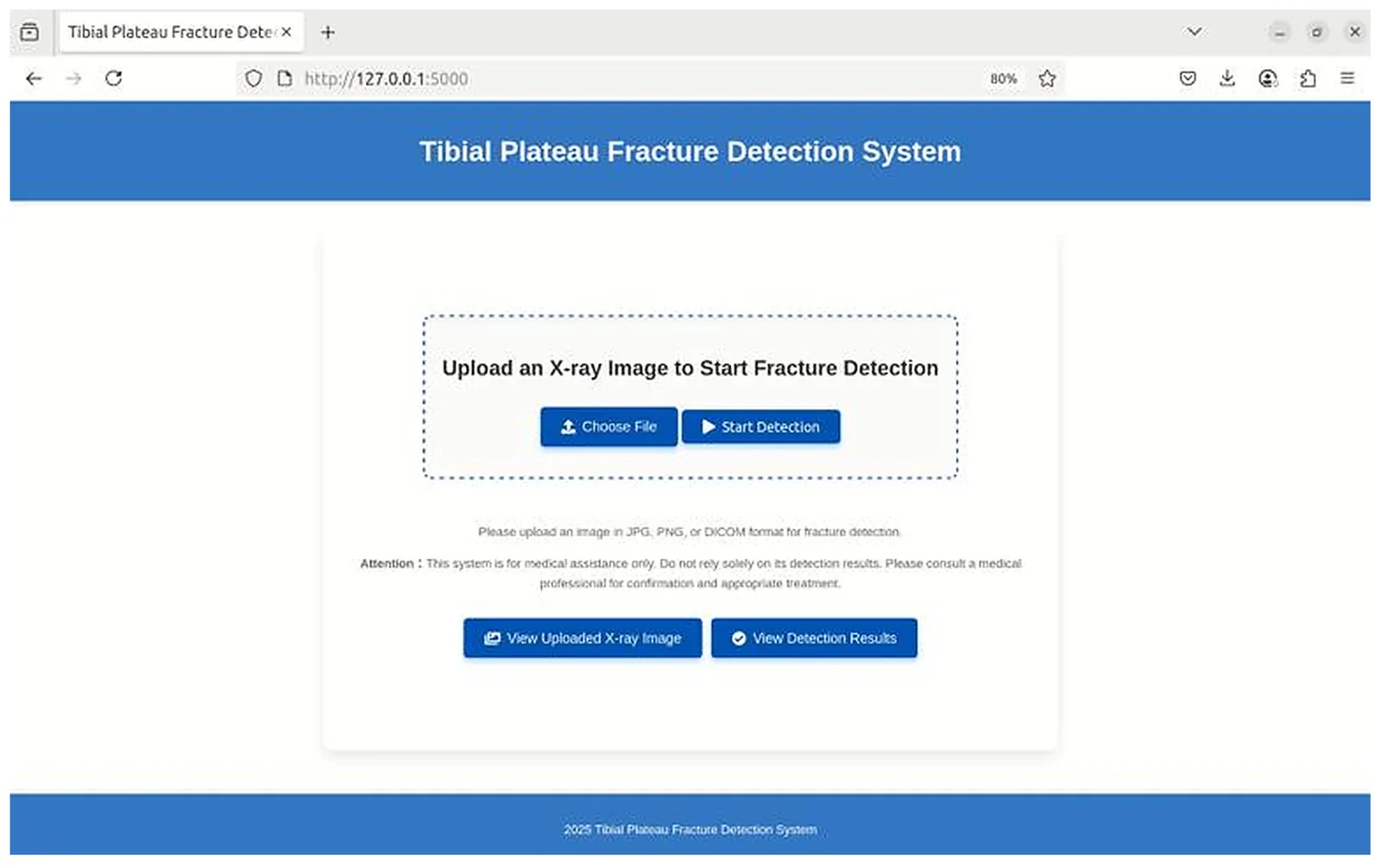
Main interface of the Tibial Plateau Fracture Detection System.

**Figure 11:**
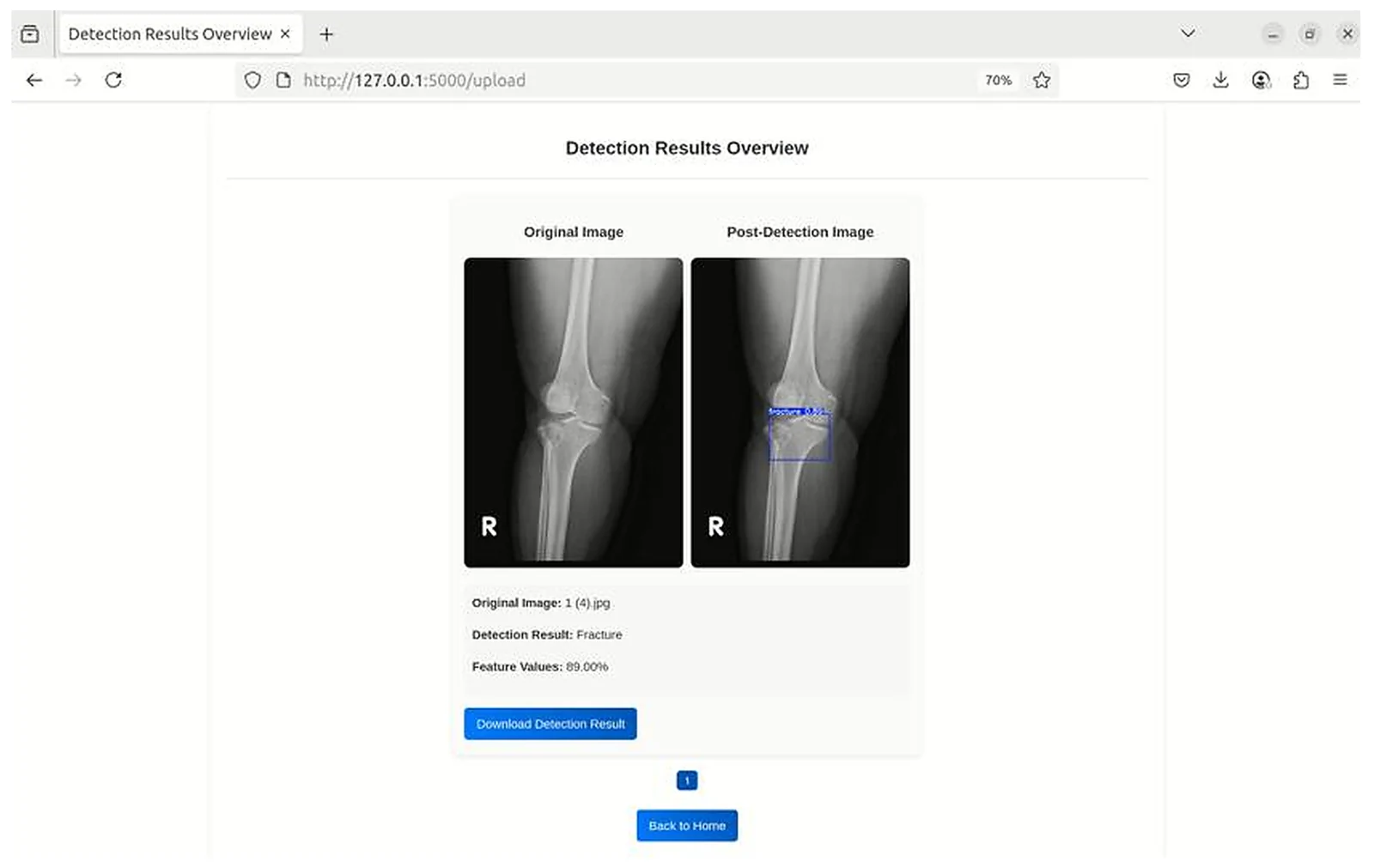
Example of a positive fracture detection result displayed on the web interface.

**Figure 12:**
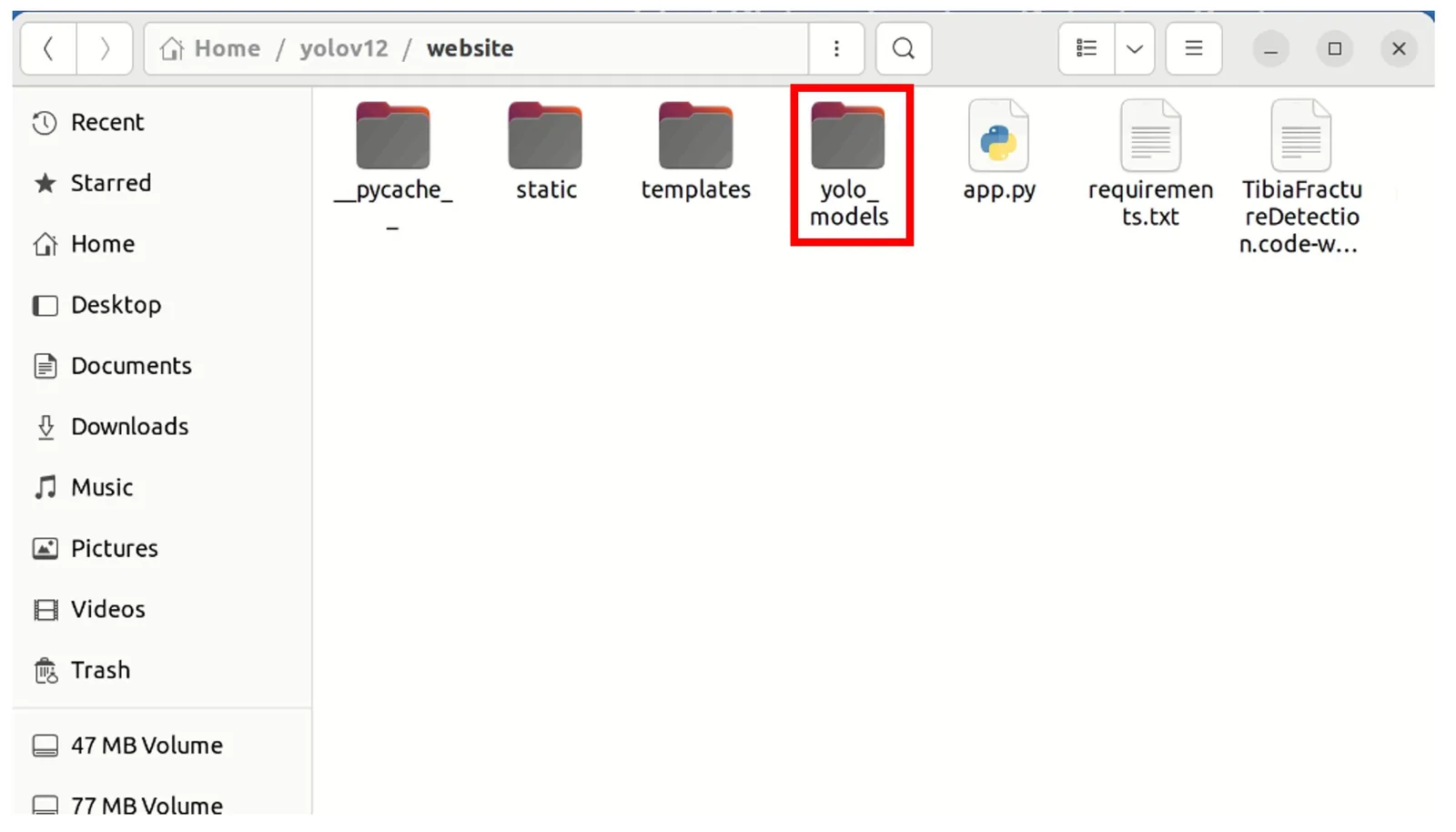
Model backend architecture for model replacement. The red box highlights the yolo_models directory, enabling seamless model updates by simply updating the corresponding weight files without modifying frontend code.

**Figure 13:**
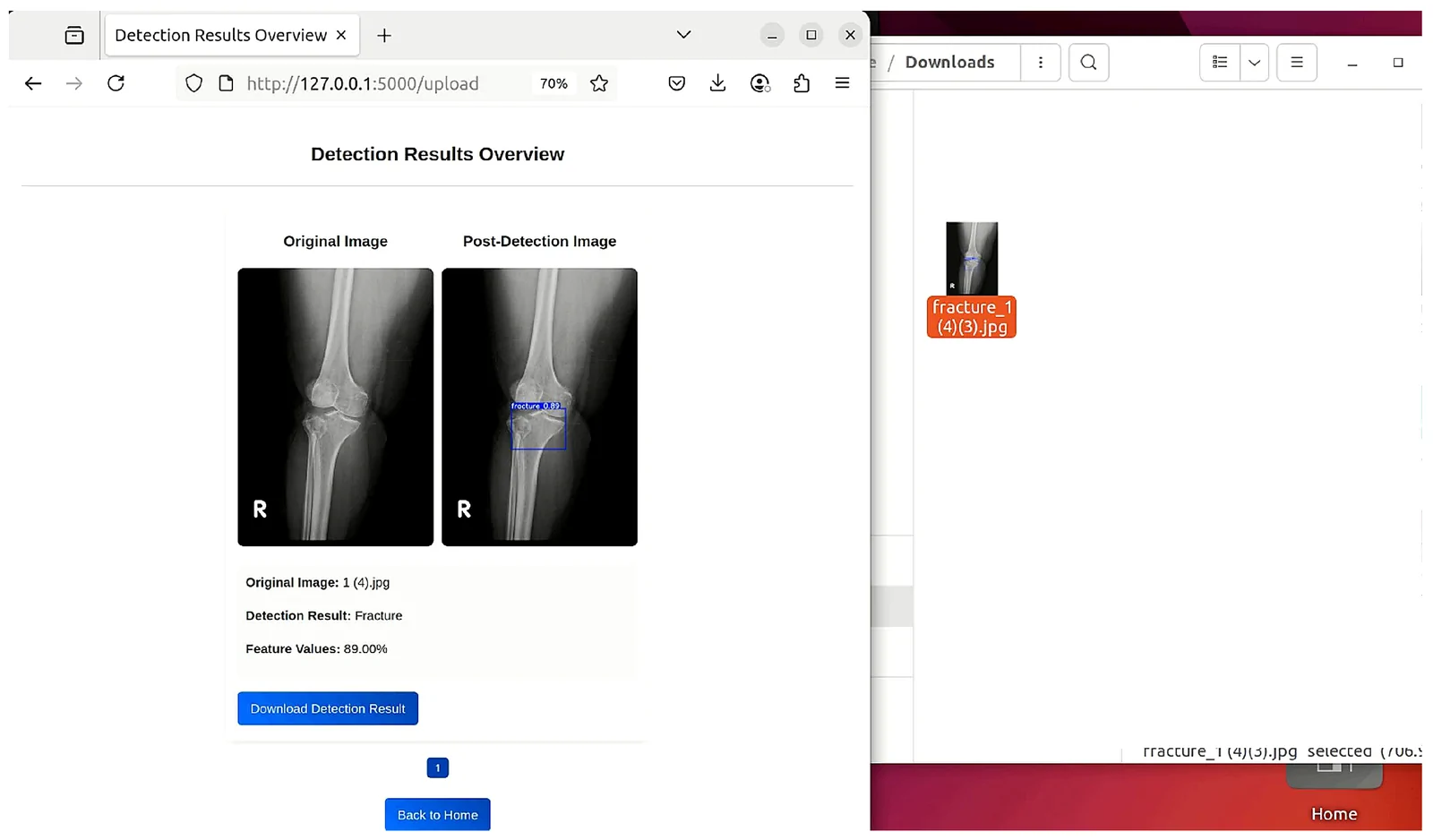
Download detection result feature, allowing clinicians to save annotated X-rays for documentation.

**Figure 14:**
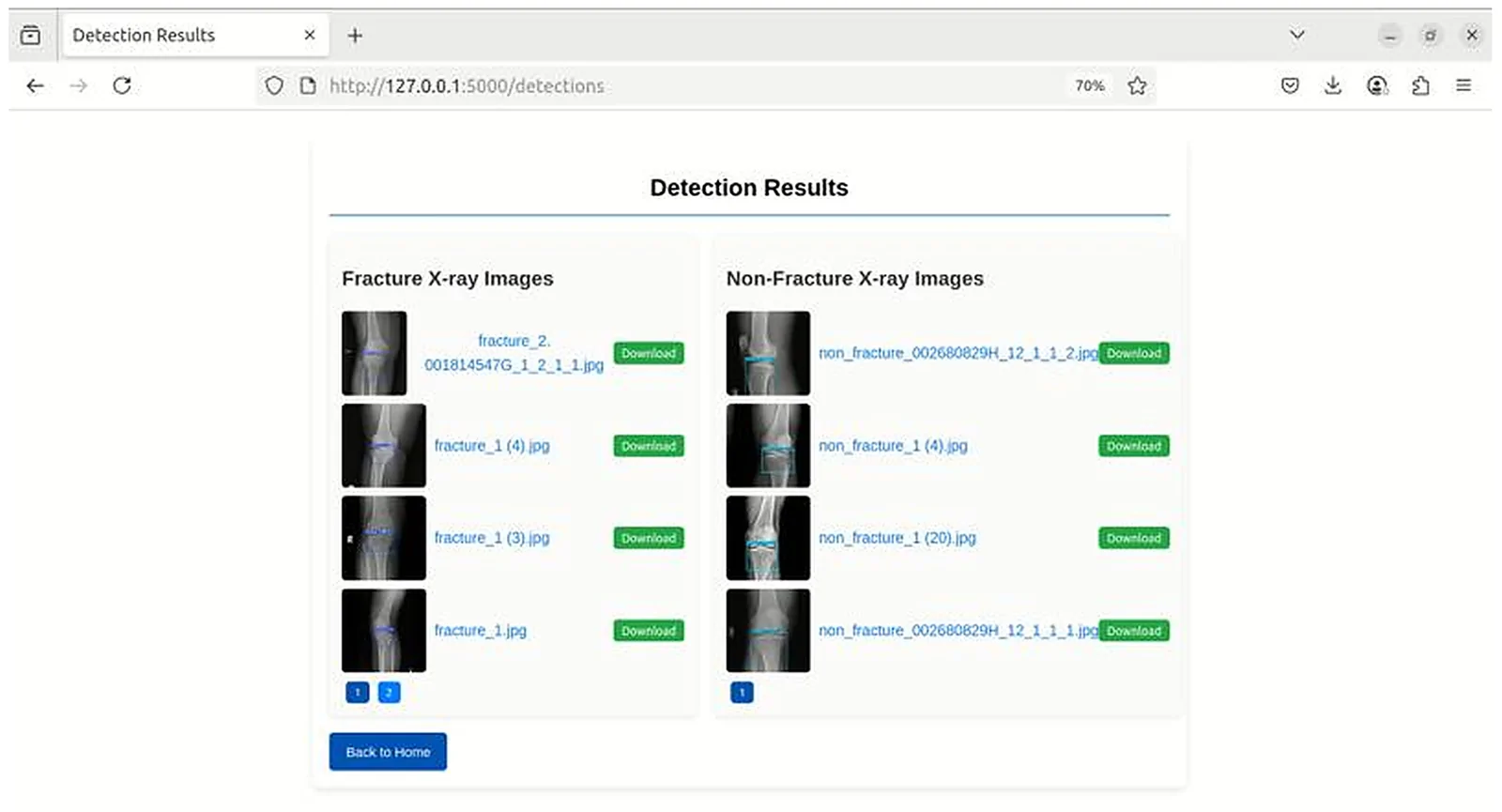
Historical Review Interface. Users can filter and retrieve past fracture and non-fracture detection records.

**Table 1:** Dataset distribution and augmentation summary.

X-Ray View	Fracture	Non-Fracture	Original Images	Augmented Images
AP View	363	383	746	1790
Lateral View	364	379	743	1785
Total	727	762	1489	3575

**Table 2:** Comparative analysis of YOLO versions during initial screening. YOLOv8 and YOLOv10 were excluded due to Background Errors (Critical Safety Risk).

Model	True Pos.	False Neg.	Background Err. *	Total Missed	Status
YOLOv8	137	8	1	9	Excluded
YOLOv9	139	7	0	7	Selected
YOLOv10	137	7	2	9	Excluded
YOLOv11	141	5	0	5	Selected
YOLOv12	139	7	0	7	Selected

* Background Err.: The model failed to detect any object (critical detection failure).

**Table 3:** Comparative evaluation metrics for YOLOv9, YOLOv11, and YOLOv12 on the test set (n = 298) with 95% confidence intervals (CIs).

Model	Accuracy [95% CI]	Sensitivity [95% CI]	Specificity [95% CI]	Precision [95% CI]	F1-Score
YOLOv9	97.65% [95.23–98.86]	95.21% [90.43–97.66]	100.00% [97.54–100.00]	100.00% [97.31–100.00]	97.56%
YOLOv11	98.32% [96.13–99.28]	96.58% [92.23–98.53]	100.00% [97.54–100.00]	100.00% [97.35–100.00]	98.25%
YOLOv12	97.32% [94.79–98.63]	95.21% [90.43–97.66]	99.34% [96.37–99.88]	99.29% [96.09–99.87]	97.20%

**Table 4:** Web Interface Validation Results (n = 109).

	Predicted Fracture	Predicted Non-Fracture
Actual Fracture (n = 50)	49	1 (FN)
Actual Non-Fracture (n = 59)	1 (FP)	58

## Data Availability

The data presented in this study are available on request from the corresponding author due to the secure environment of our institution following appropriate review and approval.
